# Bone loss in young adults with HIV following antiretroviral therapy containing tenofovir disoproxil fumarate regimen using machine learning

**DOI:** 10.3389/fphar.2025.1516013

**Published:** 2025-04-04

**Authors:** Ling Chen, Jia Tang, Leidan Zhang, Liyuan Zheng, Fada Wang, Fuping Guo, Yang Han, Xiaojing Song, Wei Lv, Wei Cao, Taisheng Li

**Affiliations:** ^1^ Department of Infectious Diseases, Peking Union Medical College Hospital, Chinese Academy of Medical Sciences and Peking Union Medical College, Beijing, China; ^2^ School of Medicine, Tsinghua University, Beijing, China; ^3^ State Key Laboratory of Complex Severe and Rare Diseases, Peking Union Medical College Hospital, Chinese Academy of Medical Science and Peking Union Medical College, Beijing, China

**Keywords:** HIV, tenofovir disoproxil fumarate, antiretroviral therapy, bone loss, machine learning

## Abstract

**Objective:**

Bone mineral density (BMD) monitoring, primarily relying on dual-energy X-ray absorptiometry (DEXA), remains inaccessible in resource-limited regions, making it difficult to promptly address bone loss in people with HIV (PWH) on long-term ART-containing TDF regimens and assess the prevalence of bone loss. Our objective is to identify the frequency of PWH experiencing bone loss after long-term ART with a TDF regimen and to develop a predictive model of HIV-infected high-risk populations containing TDF long-time ART, for providing more appropriate ART regimens for PWH in clinical practice, particularly in resource-limited settings.

**Methods:**

Our study retrospectively screened PWH under long-term follow-up at Peking Union Medical College Hospital (PUMCH) from January 2000 to August 2024. These individuals were either treatment-naive or treatment-experienced and had been on containing TDF ART regimen for over 5 years. BMD was assessed using DEXA every 1–2 years in this center. We selected predictive factors utilizing machine learning methods, including Random Forest, XGBoost, LASSO regression, and logistic regression. The results were visualized using a nomogram.

**Results:**

Our study enrolled a total of 232 PWH who have contained TDF ART regimens for more than 5 years. Twenty-five percent (58/232) of the patients experienced bone loss, primarily including osteopenia and osteoporosis. Further results showed that the LASSO regression model was the most suitable for the current dataset, based on a comparison of LASSO regression, Random Forest, XGBoost, and logistic regression models including age, gender, LPV/r, baseline CD4+ T count, baseline VL, baseline body weight, treatment-naïve TDF, ART duration, percentage of CD38+CD8+T, percentage of HLA-DR+CD8^+^ T, and CD4+/CD8+ ratio, with AUC values of 0.615, 0.507, 0.593, and 0.588, respectively. We identified age, gender, and LPV/r as the most relevant predictive factors associated with bone loss based on LASSO regression. Then the results were visualized and plotted in a nomogram.

**Conclusion:**

Our study quantified the frequency and established a nomogram based on the LASSO regression model to predict bone loss in PWH on long-term containing TDF ART. The nomogram guides identifying individuals at high risk of bone loss due to prolonged TDF exposure. Clinicians can leverage the predicted risk to design personalized ART regimens at the initiation of therapy or to switch from TDF-containing to TDF-free regimens during treatment. This approach aims to reduce the incidence of bone loss, particularly in resource-limited settings.

## Introduction

With the advancement of antiretroviral therapy (ART), the life expectancy of people with HIV (PWH) has significantly increased (Negredo et al., 2018). However, non-AIDS-defining events have become increasingly prominent, now representing major contributors to morbidity and mortality among PWH receiving suppressive ART (Maartens et al., 2014). These conditions primarily encompass cardiovascular diseases, non-AIDS-defining cancers, metabolic syndrome, liver and kidney diseases, as well as bone-related complications, such as bone loss and osteonecrosis (Dominguez-Molina et al., 2016).

Bone health in PWH is significantly influenced by a complex interplay of traditional risk factors, such as aging, comorbidities, low body mass index (BMI), smoking, alcohol abuse, and vitamin D insufficiency. Additionally, HIV-related factors, including the infection itself, low CD4 count, and antiretroviral therapy (ART) (Rey et al., 2015; Biver, 2022; Compston, 2016), further complicate this issue. Notably, certain ART medications, particularly tenofovir disoproxil fumarate and protease inhibitors (PIs), have been shown to exacerbate the loss of bone mineral density (BMD) associated with HIV infection. Research indicates that PWH can experience an additional 2%–6% reduction in BMD within the first 1–2 years of initiating ART, a rate of bone loss comparable to that observed in postmenopausal osteoporosis (McComsey et al., 2010; Han et al., 2020; Compston, 2014).

Integrase strand transfer inhibitors (INSTIs) have emerged as a new frontline option for PWH (Scarsi et al., 2020). However, tenofovir disoproxil fumarate (TDF) and protease inhibitors (PIs) continue to be the first-line ART drugs in resource-limited settings (Takou et al., 2019; Tang et al., 2023). Bone involvement is characterized by the presence of osteopenia or osteoporosis as identified through dual-energy X-ray absorptiometry (DEXA) scanning (Atencio et al., 2021; El Maghraoui and Roux, 2008). Current international practice guidelines recommend that DEXA scans be conducted for PWH aged 50 years and older (Negredo et al., 2018; Lustig et al., 2016). Despite these recommendations, access to DEXA scanning remains limited in many low-middle-income countries. While some studies have explored fracture risk prediction using mathematical models or machine learning techniques, such as FRAX and Garvan (Kanis et al., 2009; Nguyen et al., 2008; Galassi et al., 2020), these approaches may not be generalizable to PWH due to variations in population characteristics and sample size limitations. Consequently, no fracture risk prediction model specifically tailored for PWH that is available for clinical use.

This study aims to develop a predictive model for bone loss in PWH using deep learning techniques, with a particular focus on those who are either undergoing long-term tenofovir disoproxil fumarate (TDF)-based ART regimens or are about to initiate such treatment. By enabling early identification of bone loss risk in PWH, the model seeks to inform optimal ART regimen selection, especially in resource-limited settings, ultimately improving the quality of life for PWH.

## Methods

### Study population and data collection

Our study retrospectively collected data from PWH who had regular follow-ups at Peking Union Medical College Hospital (PUMCH) from January 2000 to August 2024. Inclusion criteria: ① age≥18 years; ②ART containing-TDF regimen for more than 5 years; ③ normal bone mineral density (BMD) at initiated treatment; ④ at least two bone density assessments during follow-up. Exclusion criteria: ① age <18 years; ② ART not include TDF regimens or containing-TDF regimen for less than 5 years; ③ unknown BMD or evidence of osteopenia, osteoporosis, even fractures at the start of treatment; ④ lack of BMD monitoring during follow-up; Comorbidity affecting BMD such as severe liver disease, kidney disease, thyroid disease, parathyroid disease, malignancies, wasting diseases, and PWH on anti-osteoporosis medications. We primarily collected data on the individuals’ gender, age, baseline body weight, determine the time of HIV infection diagnosis, ART regimens, ART duration, baseline CD4^+^ T count, baseline viral load (VL), baseline percentage of CD38^+^CD8^+^ T cells, baseline percentage of HLA-DR+CD8^+^ cells, CD4/CD8 ratio, and BMD results.

### Data measurement and definition

The BMD was evaluated by a dual-energy x-ray absorptiometry (DEXA) test in the center, which was rarely widespread in other medical centers due to limited medical conditions in China. The measures of lymphocyte subsets including CD4 + T count, percentages of CD38^+^CD8^+^ T cells, HLA-DR+CD8^+^ cells, CD4/CD8 ratio, and VL were adopted flow cytometry and real-time RT-PCR assay as described in previous study, respectively (Guo et al., 2021). We assessed BMD results using the World Health Organization (WHO) classification method. For men and women over 50 years, or postmenopausal women, the T-score that was obtained through DEXA scans of the spine, hip, or forearm was used: T-score of ≤−2.5 was defined as osteoporosis, and T-score between −1 and −2.5 was defined as osteopenia. For men under 50 years old and premenopausal women, the ISCD recommends using Z-scores to report BMD (Lewiecki et al., 2008), which compares an individual’s BMD to the average value of a reference population matched for age, sex, and ethnicity. A Z-score above −2.0 is considered within the normal range for the corresponding age, while a Z-score below −2.0 is defined as “below the expected range for age” (Cosman et al., 2014). Bone loss primarily includes osteopenia and osteoporosis according to the WHO guidelines or those classified as “below the expected range for age” based on the ISCD criteria in this study.

### Statistical analyses

Tabulated descriptive data were presented as frequencies. Mean with standard deviation for variables following a normal distribution and median with interquartile range (IQR) for variables not following a normal distribution. We used the t-test for parametric continuous variables, the Mann-Whitney U test for non-parametric continuous variables, and the Chi-squared test or Fisher’s exact test for categorical variables to compare the clinical characteristics of patients in different groups. The data were split into training and testing sets in a 7:3 ratio. The models evaluated include LASSO regression, logistic regression, Random Forest, and XGBoost, Receiver operating characteristic (ROC) curve analysis was performed on these models to assess the ability and the optimal cutoff value for diagnosis. The area under the ROC curves (AUC) were calculated. The most valuable variables were selected using the LASSO regression. Based on these valuable variables, a logistic regression model was constructed, and a nomogram was created based on the model. All statistical analyses were performed using SPSS 26.0 (IBM Corporation, Armonk, New York, United States) and R software version 4.4.1 and the “glmnet” package, “randomForest” package, “rms” package, and XGboost package (R Foundation for Statistical Computing, Vienna, Austria). For all tests, p < 0.05 was considered statistically significant.

## Results

### Characteristics of the study population

This study retrospectively included a total of 232 PWH who had been on long-term antiretroviral therapy regimens containing TDF, from the AIDS Treatment Center at Peking Union Medical College Hospital (PUMCH), between January 2000 and August 2024. The baseline data were summarized in Table 1. The majority of them were male, with an average age of 37.2 years and a baseline mean weight of 67.5 kg. The smoking and drinking status of the participants was not clear. The median time since the HIV infection diagnosis was 9.4 years. Their baseline median CD4^+^ T count and median viral load (VL) were 230 cells/µL and 4.7 log10 copies/mL, respectively. In addition to tenofovir disoproxil fumarate (TDF), the ART medications included lamivudine (3 TC), emtricitabine (FTC), efavirenz (EFV) 600 mg, EFV 400 mg, that has been proven to be safe and effective in the ART of HIV-infected individuals in China (Xu et al., 2021; Yang et al., 2024), nevirapine (NVP), lopinavir/ritonavir (LPV/r), raltegravir (RAL), and dolutegravir (DTG). The proportions of PWH containing EFV 600 mg and EFV 400 mg ART regimens were 61.2% and 3.9%, respectively.165 treatment-naive PWH based on TDF-containing regimens, while 67 PWH had switched to TDF-containing ART regimens. The median duration of TDF-containing ART among these individuals was 7.8 years. Twenty-five percent (58/232) of the patients experienced bone loss, with 21.6% (50/232) having osteopenia and 3.4% (8/232) having osteoporosis. 27.2% (63/232) of individuals took vitamin D. As the duration of ART increased, the cumulative incidence of bone loss showed a rising trend year by year (Supplementary Figure S1). The median time to the onset of bone loss was 5.4 years.

**TABLE 1:** Clinical characteristics of study participants.

Characteristics	Study population (N = 232)
Sex-n (%)	
Male	215 (92.7)
Female	17 (7.3)
Ages (years)-mean±SD	37.2 ± 10.2
Baseline body weight (kg)- mean ± SD	67.5 ± 11.1
Cigarette smoking	Unknown
Alcohol drinking	Unknown
Duration of HIV infection (years)-median (IQR)	9.4 (6.9,12.0)
Vitamin D-n (%)	
Yes	63 (27.2)
No	169 (72.8)
Baseline CD4^+^ T count (cells/μL)-median (IQR)	230 (76, 321)
Baseline HIV-1 RNA (copies/mL)-median (IQR)	4.7 (4.4, 5.1)
ART regimens-n (%)	
3TC + TDF + EFV 600 mg	142 (61.2)
3TC + TDF + EFV 400 mg	9 (3.9)
3TC/TDF + NVP + DTG	1 (0.4)
3TC + TDF + DTG	3 (1.3)
3TC + TDF + EFV 600 mg + RAL	1 (0.4)
3TC + TDF + LPV/r	31 (13.4)
3TC + TDF + LPV/r + DTG	1 (0.4)
3TC + TDF + LPV/r + RAL	5 (2.2)
3TC + TDF + NVP	34 (14.7)
3TC + TDF + RAL	2 (0.9)
FTC/TDF + DTG	1 (0.4)
FTC/TDF + RAL	2 (0.9)
Treatment-naïve of TDF-containing regimes-n (%)	165 (71.1)
Treatment-experienced of TDF-containing regimes-n (%)	67 (28.9)
ART duration (years)-median (IQR)	7.8 (6.2, 9.7)
Boss loss-n (%)	58 (25)
Osteopenia-n (%)	50 (21.6)
Osteoporosis-n (%)	8 (3.4)
Time of bone loss occurrence (years)-median (IQR)	5.4 (4.0, 7.2)

SD, standard deviation; kg, kilogram; IQR, interquartile range; μL, microliter; C; mL, milliliter; 3TC, lamivudine; TDF, tenofovir disoproxil fumarate; EFV, efavirenz; NVP, nevirapine; FTC, DTG, dolutegravir; RAL, raltegravir; LPV/r, lopinavir/ritonavir; ART, antiretroviral therapy.

We further compared the baseline characteristics of PWH who experienced bone loss with those who remained on TDF without any bone loss. The detailed information was depicted in Table 2. The bone loss group had a significantly higher proportion of females (15.5% vs 4.6%, p = 0.006), a higher median baseline age (43 vs 34 years, p = 0.000), and a lower baseline weight (64 ± 11.2 kg vs 69 ± 10.8 kg, p = 0.007). However, there were no significant differences between the two groups regarding baseline immune status, including CD4^+^ T cell count, CD38CD8%, HLA-DRCD8%, and CD4/CD8 ratio. Additionally, viral load and the proportion of PWH in the AIDS stage were comparable between the groups. Notably, a significantly higher percentage of PWH on ART regimens containing LPV/r were found in the bone loss group (27.6% vs 12.1%, p = 0.005). Interestingly, the duration of TDF-containing ART was shorter in the bone loss group compared to the control group (5.4 years vs 7.7 years, p = 0.000).

**TABLE 2:** Clinical characteristics of the study population experienced bone loss.

Characteristics	Control (n = 174)	Bone loss (n = 58)	p value
Sex-n (%)			
Male	166 (95.4)	49 (84.5)	0.016
Female	8 (4.6)	9 (15.5)	0.006
Ages (years)-median (IQR)	34 (27, 42)	43 (34, 48)	0.000
Baseline body weight (kg)- mean ± SD	69 ± 10.8	64 ± 11.2	0.007
Baseline CD4^+^ T count (cells/μL)-median (IQR)	237 (101, 321)	183 (43, 320)	0.197
Baseline CD38^+^CD8+/CD8 (%)-median (IQR)	76.5 (65.4, 85.6)	78.4 (59.5, 92.0)	0.449
Baseline HLA-DRCD8+/CD8 (%)-median (IQR)	61.0 (43.2, 71.8)	57.9 (45.3, 72.0)	0.997
Baseline CD4/CD8 ratio-median (IQR)	0.27 (0.12, 0.41)	0.27 (0.08, 0.42)	0.801
Baseline HIV-1 RNA (copies/mL)-median (IQR)	4.7 (4.4, 5.1)	4.7 (4.2, 5.3)	0.661
AIDS stage-n (%)	66 (37.9)	30 (51.7)	0.065
ART with LPV/r-containing regimen-n (%)	21 (12.1)	16 (27.6)	0.005
ART duration (years)-median (IQR)	7.7 (6.2, 9.8)	5.4 (4.0, 7.2)	0.000

IQR, interquartile range; kg, kilogram; SD, standard deviation, μL, microliter; HLA, human leucocyte antigen; RNA, ribonucleic acid; mL, milliliter; AIDS, acquired immune deficiency syndrome; ART, antiretroviral therapy; LPV/r, lopinavir/ritonavir.

### Predictive model construction based on deep learning

**FIGURE 1:**
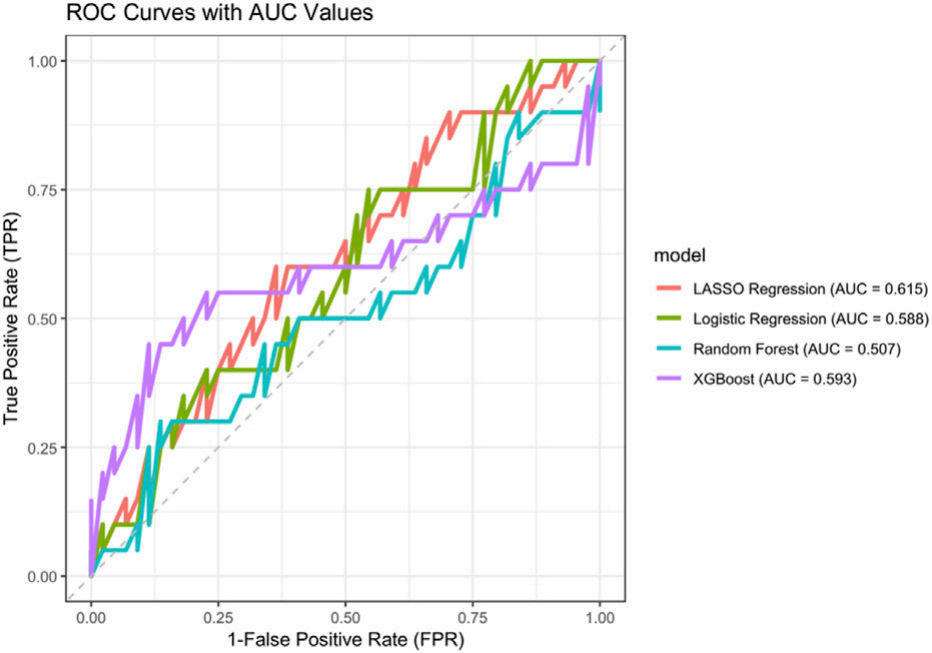
ROC curves of different machine learning models. Red: LASSO regression; green: Logistic regression; blue: Random forest model; purple: XGBoost model.

After handling the missing values, data from a total of 216 PWH were included in the study and randomly divided into training and validation sets in a 7:3 ratio. All variables including age, gender, LPV/r, baseline CD4^+^ T count, baseline VL, baseline body weight, treatment-naïve TDF, ART duration, percentage of CD38^+^CD8+T, percentage of HLA-DR+CD8^+^ T, and CD4+/CD8+ ratio showed no significant differences between the groups, except for the duration of ART (Table 3). We further compared the sensitivity and specificity of the LASSO regression, logistic regression, random forest, and XGBoost models by generating Receiver Operating Characteristic (ROC) curves. It was observed that LASSO regression had the largest area under the curve (AUC), with values of 0.615, 0.588, 0.507, and 0.593, respectively (Figure 1).

**TABLE 3:** Comparison of clinical data between training set and validation set.

Variates	Total (n = 216)	Training set (n = 152)	Test set (n = 64)	p value
Ages-mean±SD	37 ± 10.0	37 ± 10.3	37 ± 9.5	0.580
Sex-n (%)				
Male	202 (94)	145 (95)	57 (89)	0.155
Baseline body weight-mean±SD	68 ± 10.9	68 ± 10.6	66 ± 11.6	0.183
Baseline CD4^+^ T count (cells/μL)-median (IQR)	236 (94, 324)	247 (126, 334)	201 (32, 316)	0.069
Baseline HIV-1 RNA (copies/mL)	4.7 (4.3, 5.1)	4.7 (4.4, 5.1)	5 (4.2, 5.3)	0.365
Treatment-naïve of TDF-containing regimes-n (%)	161 (75)	111 (73)	50 (78)	0.432
ART with LPV/r-containing-regimen-n (%)	35 (16)	20 (13)	15 (23)	0.061
ART duration (years)-median (IQR)	7.5 (6.2, 9.6)	7.9 (6.5, 9.8)	6.7 (5.7, 8.8)	0.001
Baseline CD38^+^CD8+/CD8 (%)-median (IQR)	78.0 (66.0, 87.3)	77.6 (64.9, 86.7)	79.2 (69.9, 88.6)	0.520
Baseline HLA-HLA-DRCD8+/CD8 (%)-median (IQR)	61.4 (45.5, 72.6)	61.1 (45.5, 71.1)	61.9 (45.2, 73.3)	0.588
Baseline CD4/CD8 ratio-median (IQR)	0.27 (0.13, 0.42)	0.29 (0.14, 0.42)	0.24 (0.08, 0.41)	0.193
Bone loss- n (%)	51 (24)	31 (20)	20 (31)	0.086

SD, standard deviation; IQR, interquartile range; μL, microliter; LPV/r, lopinavir/ritonavir; RNA, ribonucleic acid; HLA, human leucocyte antigen; mL, milliliter.

**FIGURE 2:**
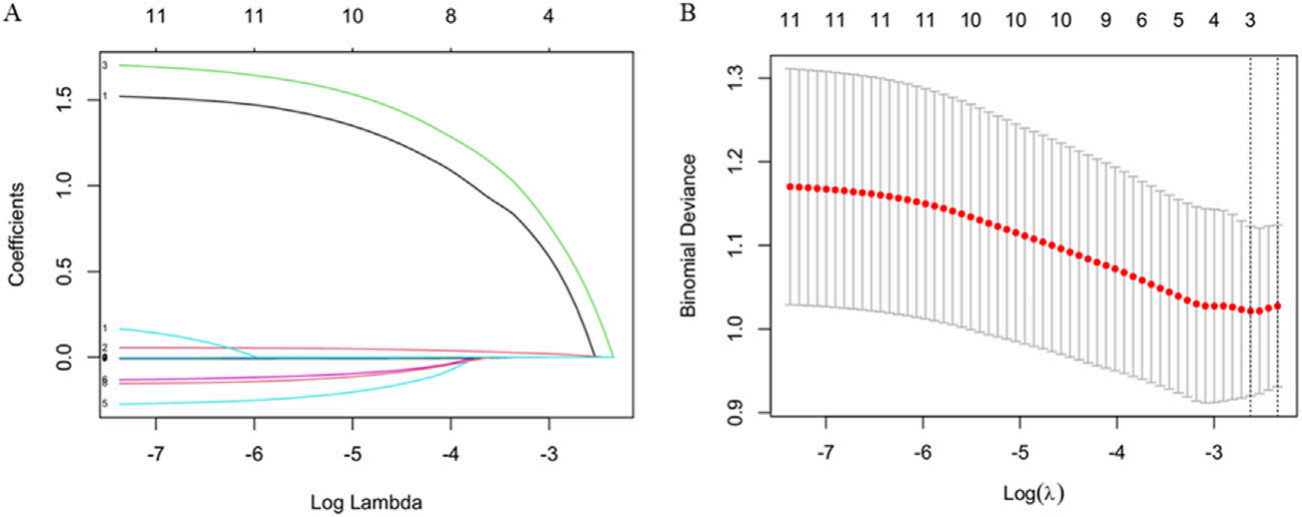
Variable selection based on LASSO regression. **(A)** The variation characteristics of the coefficient of variables **(B)** The selection process of the optimum value of the parameter λ in the Lasso regression model by cross-validation method.

Lasso regression was employed to select variables, and the variation in their coefficients is displayed in Figure 2A. A 10-fold cross-validation was performed to the penalty term, and the optimal model with the fewest variables was achieved at λ = 0.096 (Log λ = −2.35) (Figure 2B), reflecting strong model performance. Three variables including sex, age, and LPV/r (Kaletra) were selected based on LASSO regression for further logistic regression analysis (Table 4).

**TABLE 4:** The variables selected based on LASSO regression.

Variables	OR	95% CI	p value
Age	1.046	1.012–1.018	0.008
Sex	2.592	0.786–8.450	0.110
Kaletra	2.991	1.344–6.599	0.007

**FIGURE 3:**
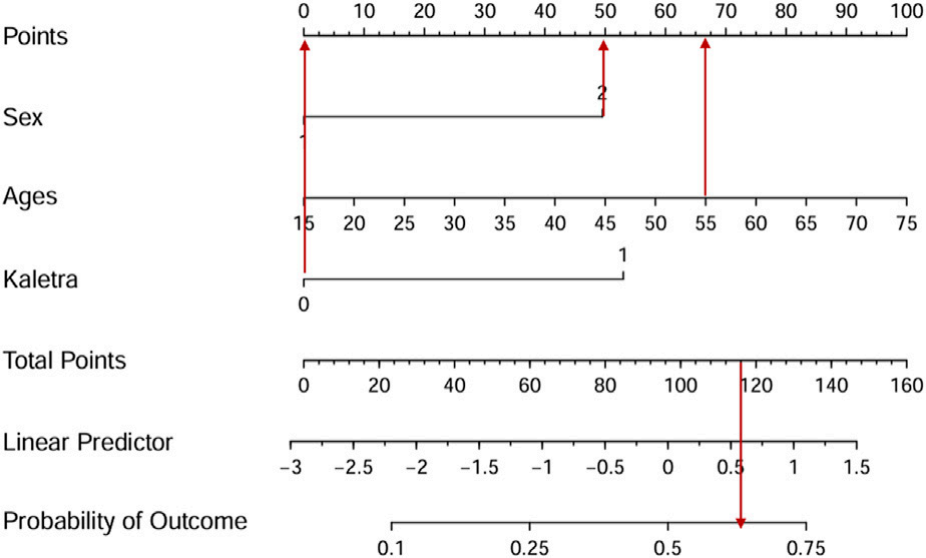
The nomogram visualizes the predictive model. For example, a 55-year-old female patient whose ART regimen includes TDF but not LPV/r has a total score of approximately 116, indicating a probability of about 62.5% for experiencing bone loss with long-time TDF use.

To facilitate clinical services, we transformed the complex mathematical model into a nomogram (Figure 3). The scores of the variables included in the model need to be summed. A vertical line is then drawn at the total score, intersecting with the line representing the clinical outcome (bone loss). For example, a 55-year-old female patient whose ART regimen includes TDF but not LPV/r has a total score of approximately 116, indicating a probability of about 62.5% for experiencing bone loss with long-time TDF use. It could be seen that nomogram was more convenient to use in clinical practice than mathematical formulas.

## Discussion

Over the past 2 decades, the development of more effective and better-tolerated anti-HIV therapies has significantly extended the long-term survival of most patients receiving treatment. This now closely aligns with that of the general population and may be comparable in a significant subset (Samji et al., 2013; Lewden et al., 2007). Osteoporosis is the most prevalent bone disease in the general population, with skeletal fragility fractures being its primary complication, leading to substantial medical, functional, and economic burdens (Yin and Falutz, 2016). Although fractures can occur at any age, individuals with HIV, both male and female, who are receiving treatment face a higher-than-expected fracture risk compared to the general population of the same age (Dong et al., 2014). The reported prevalence of low BMD in PWH varies widely in the literature, ranging from 13.9% to 88.3% (Paccou et al., 2018; Cazanave et al., 2008; Cervero et al., 2018; Shao et al., 2024; Meng et al., 2022). Specifically, ART-related bone loss is more pronounced in regimens that include TDF. A meta-analysis demonstrated that PWH on stable ART experienced a more significant decline in BMD when treated with TDF, with an annual decrease of 0.67% in the lumbar spine and 0.35% at the total hip after the first year (Starup-Linde et al., 2020). Our previous study also indicated that PWH exposed to TDF for 2 years experienced a 4.37% reduction in hip BMD compared to baseline (Guo et al., 2021). However, few studies on changes in BMD among long-term PWH undergoing TDF-containing ART regimens. A 5-year prospective study by Han et al. showed that after 60 months of TDF-containing ART, BMD decreased by 5.4% in the lumbar spine and 4% in the total hip, respectively (Han et al., 2020). However, few reports on the frequency of low BMD or fractures associated with TDF-containing ART. In our study, the frequency of low BMD among PWH on long-term TDF-based ART was approximately 25% (Table 1), which was relatively high. Moreover, with the extension of ART duration, the incidence of cumulative bone loss showed an upward trend (Supplementary Figure S1). This further suggests that in resource-limited settings, particularly where BMD measurement is not feasible, long-term TDF-containing ART regimens should be chosen with caution. In cases where suitable alternatives are unavailable, expanding the indications for free treatment with new INSTIs may be a consideration for future clinical experts.

Furthermore, we compared the clinical characteristics of PWH who experienced bone loss with those in a control group without bone-related adverse events. We found that the bone loss group had a lower pre-treatment body weight and a higher proportion of regimens including LPV/r, which were consistent with previous studies. A meta-analysis indicated that low body weight may largely account for the high prevalence of low BMD reported in PWH (Bolland et al., 2007). Katherine et al. also suggested that low body weight was more strongly negatively associated with BMD in HIV-positive persons (Kooij et al., 2015). A large number of studies have consistently concluded that TDF in combination with LPV/r results in a greater decrease in BMD and a higher risk of fracture than PWH with the unenhanced TDF group (Guo et al., 2021; Hill et al., 2018). Recent studies in China had also found that ART regimens containing LPV/r can lead to a decrease in BMD in the short term (Shao et al., 2024; Guan et al., 2021). This may be related to the fact that LPV/r increases the AUC of TDF, thereby potentially exacerbating the bone adverse events of TDF (Vitoria et al., 2016). Meanwhile, bone adverse events are also associated with gender and age, with women more likely to experience osteopenia or osteoporosis than men (Ahmed et al., 2023). Older age was also associated with a greater risk of bone loss, especially in postmenopausal women (Jaqua et al., 2022; Sharma et al., 2022). Our study also reached a consistent conclusion that although the median age of PLWH was less than 50 years (Table 2). Thus, it also potentially suggests that monitoring of BMD is equally important in PWH younger than 50 years of age, informing future adjustments to actual guidelines (Atencio et al., 2021).

Our study did not observe any differences in immune markers between individuals with and without bone loss including CD4^+^ T cell count, HIV VL and advanced disease status, although some studies had reported that the immune status of PWH may also be a potential factor influencing BMD decline after ART. A randomized trial from the START bone mineral density substudy showed that immediate ART (CD4 >500 cells/μL) resulted in greater BMD declines than deferred ART (CD4 <350 cells/μL) at the hip (−2.5% versus −1.0%, p < 0.001) and spine (−1.9% versus −0.4%, p < 0.001), further finding revealed lower CD4 count was a significant predictor of greater BMD loss at both the spine and the hip in the deferred ART group (Hoy et al., 2017). A lower CD4 count and higher HIV viral load have been associated with lower bone mass in cross-sectional studies, suggesting a role for HIV infection or the immunological response to HIV in bone loss (Kooij et al., 2015; Brown et al., 2009). However, the results of the SMART study were different. These data showed that hip BMD declined by 0.8% per year for up to 4 years of follow-up in participants who were ART-experienced at study entry, and continued using ART, but TNF-α and IL-6 increased in the intermittent ART group, and decreased in the continuous ART group. There was no evidence for an association of TNF-α, IL-6, or CD4+T cell count with any of the other BMD outcomes (Hoy et al., 2013). The variables included in these different studies vary, and as a result, the conclusions drawn may not be consistent. The more influencing factors are included, the more the conclusion will reflect the factors most closely related to BMD, just like the SMART study and this study. Immunological factors may indirectly affect bone density by influencing bone resorption and bone formation (Hoy et al., 2013).

Intriguingly, in our study, there was also a significant difference in the duration of ART with TDF between the group that experienced bone loss and the control group, where the duration was rather longer (Table 2). This seemed to contradict the finding in our study that the cumulative incidence of bone adverse events was higher as the duration was longer. One possible explanation is that the occurrence of bony adverse events with long-term use of TDF regimens occurs only in specific PWH, although most studies consider BMI or body weight, age, gender, etc., as risk factors for low BMD. In actual clinical practice, some postmenopausal HIV-infected women had not experienced bone loss with TDF-containing ART regimens. It was especially important to early identification of people who may experience bone loss. Our study compared the ROC of different machine models and found that the AUC curve was still the largest for LASSO regression. Further, three variables, age, sex and LPV/r, were selected based on LASSO regression brushing to be most associated with clinical resolution of bone loss. Finally, our results were visualized thought a nomogram. Lasso regression was superior to univariate analysis and can resolve multicollinearity between variables. In this study, we developed a nomogram based on a Lasso-logistic regression model, which can help clinical practitioners to predict PWH who develop low BMD with TDF, especially in low-middle-income countries where BMD monitoring was not available, to further optimize the treatment regimen, such as switch TDF to tenofovir alafenamide (TAF). Many clinical trials reported that switching from a TDF-containing regimen to one containing TAF, has been associated with BMD increases as measured by DEXA (Maggiolo et al., 2019; Orkin et al., 2017) and to inform the future expansion of the indications for new first-line INSTIs.

Our study also had several limitations that should not be ignored. Firstly, it was a retrospective investigation with inevitable retrospective bias. Meanwhile, this was a single-centre study with a relatively small sample size, limiting the generalizability of the findings. Furthermore, older PWH TDF-containing ART regimes were not included in our model, meaning that changes in bone mineral density (BMD) could not be predicted for PWH aged 60 years or older. However, in older patients, experienced clinicians considering bone adverse events would also try to avoid TDF-containing ART regimens. Besides, PWH (People with HIV) tend to be younger in this study, with 25% of them under the age of 50 showing bone loss. This may be related to unhealthy lifestyle habits such as smoking, drinking, lack of exercise, and high sugar and salt intake. However, we lack this data and cannot further include these factors for a comprehensive evaluation. An even more flawed point was that this study model did not include PWH who were not containing TDF ART as a control or healthy control, and it did not identify specific subgroups of PWH, which further limits the generalizability of the findings. And the AUC value is relatively low, it may reflect the complexity and heterogeneity of the data itself. To strengthen our conclusions, We will continue to optimize the model and explore additional factors that may affect its performance in future studies.

## Conclusion

Our study found that long-term ART (more than 5 years) with TDF-containing regimens is associated with a relatively high frequency of bone loss, including osteopenia and osteoporosis. We also developed a predictive model for bone loss using a LASSO-penalized logistic regression approach, which we visualized with a nomogram. This model can assist clinicians in optimizing ART regimens, such as discontinuing TDF and LPV/r in favor of TDF/LPV/r-free alternatives, or using TAF or EFV 400 mg, especially in resource-limited areas where BMD monitoring may not be available.

## Data Availability

The original contributions presented in the study are included in the article/Supplementary Material, further inquiries can be directed to the corresponding author.
