# Supplementary figures and images for: Bone loss in young adults with HIV following antiretroviral therapy containing tenofovir disoproxil fumarate regimen using machine learning
Source: Front Pharmacol. 2025 Apr 4;16:1516013. doi: 10.3389/fphar.2025.1516013 (PMC12006115; doi:10.3389/fphar.2025.1516013)

**
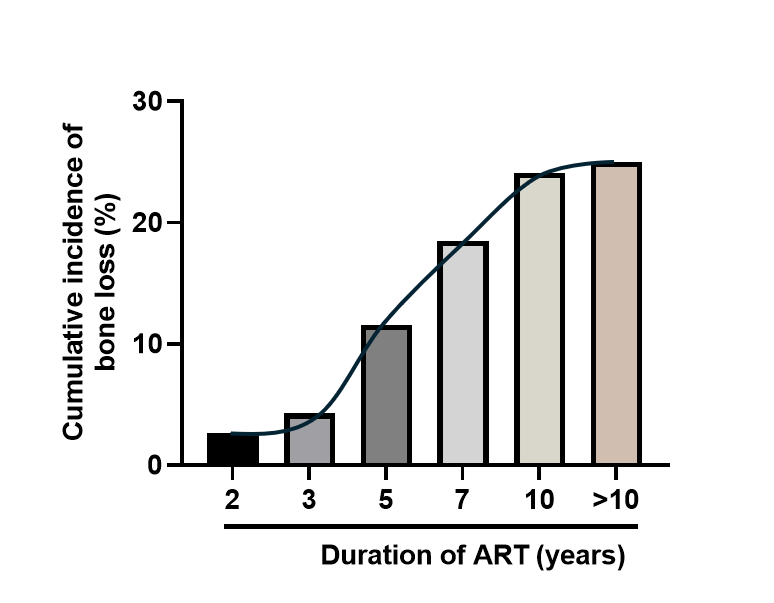
**

**Supplementary figure 1** The cumulative incidence of bone loss over time.

Supplement: Supplementary file 1 [file Supplementaryfile1.docx]
